# Microbial Degradation of Citric Acid in Low Level Radioactive Waste Disposal: Impact on Biomineralization Reactions

**DOI:** 10.3389/fmicb.2021.565855

**Published:** 2021-04-28

**Authors:** Natalie Byrd, Jonathan R. Lloyd, Joe S. Small, Frank Taylor, Heath Bagshaw, Christopher Boothman, Katherine Morris

**Affiliations:** ^1^Department of Earth and Environmental Sciences, Research Centre for Radwaste Disposal and Williamson Research Centre, The University of Manchester, Manchester, United Kingdom; ^2^National Nuclear Laboratory, Warrington, United Kingdom; ^3^Low Level Waste Repository Ltd., Seascale, United Kingdom; ^4^School of Engineering, The University of Liverpool, Liverpool, United Kingdom

**Keywords:** anaerobic biodegradation, low level radioactive waste, biodegradation, bioreduction, high pH, alkaline, citric acid, complexing agent

## Abstract

Organic complexants are present in some radioactive wastes and can challenge waste disposal as they may enhance subsurface mobility of radionuclides and contaminant species via chelation. The principal sources of organic complexing agents in low level radioactive wastes (LLW) originate from chemical decontamination activities. Polycarboxylic organic decontaminants such as citric and oxalic acid are of interest as currently there is a paucity of data on their biodegradation at high pH and under disposal conditions. This work explores the biogeochemical fate of citric acid, a model decontaminant, under high pH anaerobic conditions relevant to disposal of LLW in cementitious disposal environments. Anaerobic microcosm experiments were set up, using a high pH adapted microbial inoculum from a well characterized environmental site, to explore biodegradation of citrate under representative repository conditions. Experiments were initiated at three different pH values (10, 11, and 12) and citrate was supplied as the electron donor and carbon source, under fermentative, nitrate-, Fe(III)- and sulfate- reducing conditions. Results showed that citrate was oxidized using nitrate or Fe(III) as the electron acceptor at > pH 11. Citrate was fully degraded and removed from solution in the nitrate reducing system at pH 10 and pH 11. Here, the microcosm pH decreased as protons were generated during citrate oxidation. In the Fe(III)-reducing systems, the citrate removal rate was slower than in the nitrate reducing systems. This was presumably as Fe(III)-reduction consumes fewer moles of citrate than nitrate reduction for the same molar concentrations of electron acceptor. The pH did not change significantly in the Fe(III)-reducing systems. Sulfate reduction only occurred in a single microcosm at pH 10. Here, citrate was fully removed from solution, alongside ingrowth of acetate and formate, likely fermentation products. The acetate and lactate were subsequently used as electron donors during sulfate-reduction and there was an associated decrease in solution pH. Interestingly, in the Fe(III) reducing experiments, Fe(II) ingrowth was observed at pH values recorded up to 11.7. Here, TEM analysis of the resultant solid Fe-phase indicated that nanocrystalline magnetite formed as an end product of Fe(III)-reduction under these extreme conditions. PCR-based high-throughput 16S rRNA gene sequencing revealed that bacteria capable of nitrate Fe(III) and sulfate reduction became enriched in the relevant, biologically active systems. In addition, some fermentative organisms were identified in the Fe(III)- and sulfate-reducing systems. The microbial communities present were consistent with expectations based on the geochemical data. These results are important to improve long-term environmental safety case development for cementitious LLW waste disposal.

## Introduction

Low level radioactive waste (LLW) comprises more than 90% of the global radioactive waste inventory by volume (IAEA, 2018). LLW production in countries with significant nuclear facilities is forecast to continue rising. In particular, early nuclear nations (e.g., United Kingdom, United States, France, and Russia) will produce large quantities of LLW during decommissioning and remediation of their substantial legacy facilities (IAEA, 2018). In the United Kingdom, solid LLW is estimated to reach 1.6 million m^3^ by 2125 (NDA, 2016). Worldwide, LLW inventories contain less than 2% of the radioactivity present in total radioactive waste inventories. Typically this includes significant quantities of both short-lived, high specific activity and long-lived, low specific activity radionuclides alongside various other components such as cementitious materials and polycarboxylic acids used as decontamination agents. The radionuclide loading of LLW implies that long term disposal strategies are needed for its safe management (IAEA, 2009) and inherent to this is an understanding of the behavior of these waste forms with decontamination agents.

Organic decontaminants are the principal source of complexing agents disposed of within the UK’s LLWR (LLW Repository Ltd., 2011b); categorized as either polycarboxylic (e.g., citric and oxalic) or aminopolycarboxylic [e.g., ethylenediaminetetraacetic acid (EDTA), nitrilotriacetic acid (NTA)] acids. The metal complexing capacity of these compounds is essential in their application as decontamination agents in the fuel cycle. Simultaneously, their behavior as multidentate ligands may solubilize radionuclides and raises potential challenges with disposal. Although solidification (in a cement or plastic matrix; Brownstein, 1991) or pretreatment (e.g., by microbial degradation; Gorbunova et al., 2015; Tregubova et al., 2016, 2017) of liquid radioactive wastes prior to disposal should mitigate this to an extent, contact with water can lead to leaching of both organic chelating agents and metals into the near field (McIsaac et al., 1992; Akers et al., 1994a, b). Radioactive waste repositories strictly monitor and, in some cases, control quantities of organic complexants in waste consignments, and whilst there is usually a strict capacity limiting disposal volumes of aminopolycarboxylic acids, there is often no such capacity for the disposal of polycarboxylic acids, which are assumed to be biodegradable. Citric acid is used extensively in nuclear decontamination (Murray, 1986). Citric acid is also ubiquitous in nature and at neutral pH, citrate is able to support various anaerobic microbial metabolisms including fermentation (Starrenburg and Hugenholtz, 1991), denitrification (Francis et al., 2000; Boltyanskaya et al., 2007; Kim et al., 2007; Xu et al., 2007; Fox et al., 2015), metal reduction (Switzer Blum et al., 1998, 2016; Risso et al., 2009) and sulfate reduction (Yang et al., 2006; Gámez et al., 2009; Stams et al., 2009). Additionally, citrate can enhance Fe-bioavailability to microbes (Sandy and Butler, 2013).

Given the requirement for long term disposal strategies for LLW a multi-barrier concept is adopted with emphasis on the chemical and physical barriers of the waste and engineered features of the repository near field. Briefly, waste is compacted and grouted into iron or steel drums, before placing into steel shipping containers (ISO containers) which are grouted into place within a concrete repository vault (Finster and Kamboj, 2011). This approach is used at UK surface disposal facilities including the LLW Repository (LLWR) and Dounreay. Similar surface facilities are operational at Centre de la Manche and Centre de l’Aube (France), El Cabril (Spain) and sites at Barnwell, Clive, Hanford and Oak Ridge (United States) (Finster and Kamboj, 2011). Upon reaching capacity, the surface repositories will be capped and sealed, moving from the operational to the post-closure phase (IAEA, 2002). Eventually, water ingress and saturation will occur and anaerobic conditions will develop, driven by steel corrosion and microbial activity (IAEA, 2002). The use of cement based materials both to encapsulate waste, and in repository engineering, will generate alkaline conditions in the near field (Berner, 1992). Ultimately, an anaerobic, high pH, nutrient rich, reducing environment is expected to be generated within repositories, where microbial activity may play an important role in waste degradation (West and McKinley, 1983; Humphreys et al., 1997; LLW Repository Ltd., 2011b; Small et al., 2011). The bulk pH in the UK LLWR vault disposal system is estimated to be approximately 11 due to buffering with cement grout, but with localized niches of lower pH expected due to heterogeneity (Small et al., 2011).

In spite of the alkaline pH expected within a typical LLW repository post closure, research over the past decade has highlighted that microorganisms have the potential to colonize high pH wasteforms, particularly intermediate level waste (ILW, Rizoulis et al., 2012). Given that LLW comprises a wider variety of components (LLW Repository Ltd., 2011a, 2016), including large organic and putrescible waste fractions, bacteria are even more likely to colonize these wasteforms. Additionally, anaerobic microorganisms have been shown to function in a variety of natural high pH environments such as soda lakes (Switzer Blum et al., 1998; Zhilina et al., 2005; Zavarzina et al., 2006; Sorokin and Muyzer, 2009) and serpentinising systems (Suzuki et al., 2014; Rizoulis et al., 2012). Alkaliphiles are also present in anthropogenically generated high pH environments, including in sediments from former lime working sites (Burke et al., 2012; Rizoulis et al., 2012; Charles et al., 2015; Rout et al., 2015b; Smith et al., 2016), chromite ore processing residue (COPR; Whittleston et al., 2011; Fuller et al., 2014), and borax leachate ponds (Ye et al., 2004).

Under experimental conditions representative of cementitious ILW repositories, anaerobes have been shown to exploit a variety of terminal electron acceptors (TEAs) at pH 10–11. When acetate and lactate are used as electron donors, TEAs utilized include: nitrate, Fe(III) and sulfate up to pH 11 (Rizoulis et al., 2012) and these are also key TEAs of interest in LLW. Oxyanions, including nitrate and sulfate, are ubiquitous within the nuclear fuel cycle and during fuel reprocessing, and may also be introduced to the facility via water ingress through agricultural runoff or sea water (Ashbrook, 1986; Naylor et al., 1986; K’Zhero, 1997; Albrecht et al., 2013; Safonov et al., 2018). Redox active metal TEAs such as Fe(III) are also typically abundant in LLW. TEA utilization has been shown to follow a thermodynamically predictable succession of nitrate > Fe(III)-citrate > Fe(III)-oxyhydroxide > sulfate, even at high pH where reductive activity declines as the pH increases and/or substrate bioavailability becomes limited (Rizoulis et al., 2012; Bassil et al., 2015b). Through this cascade of terminal electron accepting processes, the Gibb’s free energy yield decreases; predictions and previous results show sulfate reduction becomes severely limited at pH values above 10 (Rizoulis et al., 2012). Other redox active metals, including radionuclides such as U(VI) and Np(V), have been shown to undergo enzymatic and also indirect reduction reactions at elevated pH, when Fe(III)-reducing conditions are established (e.g., Williamson et al., 2013, 2014, 2015). This clearly emphasizes the need to better understand the influences on, and limits of, high pH Fe biogeochemistry in these systems.

Recently, utilization of a variety of organic electron donors derived from cellulose and plastic wastes, including isosaccharinic acid (ISA; Bassil et al., 2015b; Rout et al., 2015a), gluconate and phthalate plasticizers has also been shown up to pH 10 (Bassil and Lloyd, 2018; Nixon et al., 2018). Utilization of ISA, a strong complexant for some radionuclides and metals, as an electron donor under alkaline conditions, is a particular interest to the hyperalkaline pH conditions (pH > 12) developed in ILW that promote ISA formation (Bassil et al., 2015b). However, in the case of the lower pH (pH 11) conditions expected for LLW disposal, ISA becomes less significant than the aminopolycarboxylic and polycarboxylic acids present in the wastes due to both reduced production of ISA and its potential for biodegradation at pH 11 (LLW Repository Ltd., 2011a).

Overall, little information is available regarding citrate utilization as an electron donor under conditions of relevance to surface LLW disposal (i.e., up to pH 11). Furthermore, the upper pH limits of anaerobic citrate metabolism are not clearly defined. This study aims to establish the upper pH limit for anaerobic citrate degradation via fermentation and for the key terminal electron acceptors relevant to LLW disposal: nitrate, Fe(III) and sulfate. In turn, this will inform safety cases for LLW disposal and provide direct information on the likely behavior of citrate in these complex, heterogeneous, and critical environmental protection facilities.

## Materials and Methods

In order to establish the upper pH limits and rates of anaerobic citrate degradation, microcosm experiments were set up under anaerobic conditions testing for citrate degradation via fermentation, (15 mM citrate only) or by examining the citrate mediated reduction of nitrate (5 mM citrate, 30 mM NaNO_3_), Fe(III) [1 mM and 15 mM citrate, 18 and 15 mM Fe(III)] or sulfate (15 mM citrate, 15 mM Na_2_SO_4_) as the key terminal electron acceptor. Microcosms were prepared at pH 10, 11, and 12 prior to their inoculation with sediment (5% w/v). All microcosms were set up and sampled anaerobically (N_2_ headspace) and incubated at 20°C in the dark for the duration of the experiments and suite of geochemical, mineralogical and microbiological analysis was undertaken on samples extracted throughout bioreduction. Geochemical modeling using PHREEQC (Parkhurst and Appelo, 2013), with the Thermochimie database (version 9b; Giffaut et al., 2014), was used to aid in planning experiments and interpretation of data.

### Sediment

Sediment inoculum was collected from, Harpur Hill in Derbyshire, United Kingdom, which is a well characterized legacy lime works (Rizoulis et al., 2012; Milodowski et al., 2013). Harpur Hill sediments provide a good analog for sediments expected within the near field of a cementitious repository, owing to their high pH and Ca content. Diverse communities of alkaliphiles have been observed at Harpur Hill, capable of carrying out a range of biogeochemical processes (Burke et al., 2012; Rizoulis et al., 2012; Smith et al., 2016), including when radionuclides such as U and Np are present (Williamson et al., 2014, 2015). Indeed, this previous work has indicated that bacterial communities at Hapur Hill evolved relatively quickly to survive and proliferate in the high pH and Ca rich environment. Similar evolution is expected in LLW repositories post closure, and thus, these sediments provide a suitable analog for a cementitious LLW repository. Sediments were typically used within 8 weeks of collection.

### Microcosm Experiments

Anaerobic microcosms were set up in triplicate in glass serum bottles containing: 100 mL medium (9.4 mM NH_4_Cl, 4.3 mM K_2_HPO_4_, 4 mM NaHCO_3_, 0.025 g L^–1^ yeast extract; Lovley et al., 1984), 5 g of sediment inoculum and trisodium citrate (Na_3_C_6_H_5_O_7_) as the electron donor. For the fermentation experiment 15 mM of citrate was added. In other experiments, the relevant electron acceptors and citrate were included as follows for nitrate-reducing (30 mM NaNO_3_ and 5 mM trisodium citrate) and sulfate-reducing (15 mM Na_2_SO_4_ and 15 mM trisodium citrate) experiments. Two sets of Fe(III)-reducing experiments were set up—one with low citrate concentration (18 mM ferrihydrite and 1 trisodium citrate) and one with high citrate concentration (15 mM ferrihydrite and 15 mM trisodium citrate), throughout these will be referred to as the “low” and “high” citrate systems. The inclusion of high and low citrate systems in the Fe(III)-reducing experiments was to explore the effects of stoichiometry and of citrate complexation of Fe(III) on citrate biodegradation. Triplicate experiments were adjusted to pH 10, 11, and 12 using NaOH, before 5 g of sediment inoculum was added. In some experiments, addition of the sediment inoculum slightly altered the microcosm pH. Notably, this affected the high-citrate Fe(III) experiments and here, initial pH values were recorded as pH 10.2, 10.8, and 11.7, and the “pH 11” experiment of the low citrate system which was initiated at pH 10.6. Heat-sterilized experiments, or no electron donor controls were set up in parallel.

### Geochemical Analyses

After inoculation, sediment slurry was extracted at selected time points under anaerobic conditions. The pH and Eh were measured using a Denver Instrument digital meter and Fisherbrand FB68801 electrode, calibrated before measuring each time point using pH 7, 10, and 12 buffers (Thermo Fisher Scientific). Concentrations of anionic species (nitrate, sulfate, citrate, and selected volatile fatty acids) were measured using a Dionex ICS5000 with appropriate standards (Sigma-Aldrich).

The bioavailable Fe(II) and total bioavailable Fe concentration of the sediment slurry were measured using the ferrozine assay (Lovley and Phillips, 1987). Briefly, a small aliquot of homogenized sediment slurry was added to 0.5 N HCl and digested for 1 h in the dark. An aliquot of digested sample was added to a clean quartz cuvette containing ferrozine solution and left to develop for 1 min before measuring absorbance at 562 nm (the pH of this solution was always between pH 4 and 10 to ensure stability of the Fe(II)-ferrozine complex (Stookey, 1970). Subsequently, hydroxylamine hydrochloride was added to sample digests and left to reduce Fe(III) for a further hour. These reduced sample digests were then re-measured at 562 nm. The method was calibrated for each time point using iron sulfate standards of known concentration.

### Mineralogical Characterization

Powder X-ray diffraction (XRD) and Transmission Electron Microscopy (TEM) with Energy Dispersive X-Ray Analysis (EDAX) and Selected Area Electron Diffraction (SAED), were used to characterize the solids from Fe(III)-reducing experiments; anaerobic conditions were maintained during sample preparation. To prepare samples a magnet was used to separate the dark colored, magnetic Fe-phase from the sediment inoculum in the microcosms. Aliquots of the magnetically separated material were then prepared on either a clean glass slide for XRD or a gold grid with a holey carbon film for TEM, and left to dry in the anaerobic cabinet for at least 24 h. For XRD analysis slides were placed in an anaerobic sample holder and analyzed using a Bruker D8 Advance. XRD conditions were as follows: Cu K α_1_ X-rays at 5–70 degrees, 0.02 degree step size at 0.5 s per step. Crystal patterns were matched using Eva v14 against standards from the International Centre for Diffraction Data database. TEM imaging was performed using a JEOL 2100+ fitted with a LaB_6_ source running at 200 kV. Images were collected on a Gatan RIO camera and EDAX analysis was performed using an Oxford X-Max 65T EDS detector and data analyzed using Aztec software.

### Microbial Community Analysis

#### DNA Extraction

A DNeasy PowerLyzer PowerSoil Kit (Qiagen, Manchester, United Kingdom) was used to extract DNA from 300 μL of sediment slurry. Sediment slurry from triplicate samples was pooled into one sample for each condition (e.g., a “pH 10, nitrate-reducing endpoint sample”) prior to extraction, as the DNA yield from single samples typically proved too low for straightforward analysis. Extracted 16S rRNA gene fragments were amplified through the Polymerase Chain Reaction (PCR) which was performed using 8F (5′-AGAGTTTGATCCTGGCTCAG-3′) primers, and 1492R (5′-TACGGYTACCTTGTTACGACTT-3′) primers (Lane, 1991). After amplification via PCR, the DNA was stained and placed in an agarose gel and separated by electrophoresis. Stained DNA was observed under UV light, and the target ∼1,500 base pair products identified by assessment against a ladder of varying lengths of DNA fragments. Experimental controls were included to check for contamination of reagents.

#### 16S rRNA Gene Sequencing

The Illumina MiSeq platform (Illumina, San Diego, CA, United States) was used to sequence PCR amplicons of 16S rRNA genes, targeting the V4 hyper variable region (forward primer, 515F, 5′-GTGYCAGCMGCCGCGGTAA-3′; reverse primer, 806R, 5′-GGACTACHVGGGTWTCTAAT-3′) for 2 × 250-bp paired-end sequencing (Illumina) (Caporaso, 2011; Caporaso et al., 2012). The Roche FastStart High Fidelity PCR System (Roche Diagnostics Ltd., Burgess Hill, United Kingdom) was used to amplify PCR products in 50 μL reactions under the following conditions: initial denaturation at 95°C for 2 min, followed by 36 cycles of 95°C for 30 s, 55°C for 30 s, 72°C for 1 min, and a final extension step of 5 min at 72°C. PCR products were purified and normalized to ∼20 ng each using the SequalPrep Normalization Kit (Thermo Fisher Scientific, Loughborough, United Kingdom). The PCR amplicons from all samples were pooled in equimolar ratios. The run was completed using a 4 pM sample library spiked with 4 pM PhiX to a final concentration of 10% following the method of Schloss and Kozich (Kozich et al., 2013). Raw sequences were divided into samples by barcodes (up to one mismatch was permitted) using a sequencing pipeline. Quality control and trimming was performed using Cutadapt4, FastQC5, and Sickle6. MiSeq error correction was performed using SPADes7. Forward and reverse reads were incorporated into full-length sequences with Pandaseq8. Chimeras were removed using ChimeraSlayer9, and operational taxonomic units (OTUs) were generated with UPARSE10. OTUs were classified by Usearch11 at the 97% similarity level, and singletons were removed. Rarefaction analysis was conducted using the original detected OTUs in Qiime12. The taxonomic assignment was performed by the RDP classifier (Caporaso, 2011; Kozich et al., 2013). Again, experimental controls were included to check for background contamination.

## Results and Discussion

Results from anaerobic microcosms are presented in order of nitrate-reducing, Fe(III)-reducing, sulfate-reducing experiments followed by the microbial community analysis data. The fermentation experiments did not show any changes in aqueous geochemistry or visual appearance—this implied that citrate was not fermented in the absence of an electron acceptor (Supplementary Figure 1).

### Nitrate-Reducing Conditions

**FIGURE 1:**
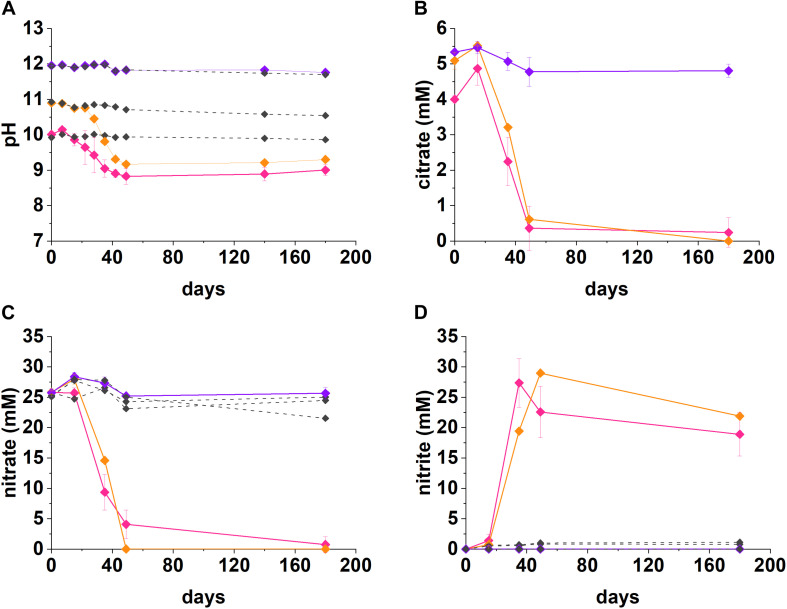
Geochemical data for nitrate-reducing experiments. The pH **(A)**, citrate **(B),** nitrate **(C),** and nitrite **(D)** data from anaerobic, high pH microcosms incubated for up to 200 days at 20°C in the dark are presented (pH 10—pink, pH 11—orange, pH 12—purple, no-citrate control—dashed gray). The errors are shown and represent 1 SD on triplicate measurements.

Under denitrifying conditions, >95% of the citrate was degraded at both pH 10 and pH 11 over 50 days, as shown in Figure 1. The maximum citrate degradation rate during denitrification was between 4.4 and 4.9 mM citrate removal in 49 days. The complete oxidation of citrate to CO_2_, as shown in Equations 1 and 2, was assumed as no organic degradation products, such as acetate or formate, were detected. Alongside this, there was a decrease in solution pH. In the pH 10 system the pH decreased to 8.8, and in the pH 11 system the pH decreased to 9.2. This mild acidification suggested full oxidation of citrate to carbonic acid followed by its subsequent dissolution and resultant acidification of the batch experimental system. The rates of nitrate reduction observed were 0.44 mM day^–1^ at pH 10 and 0.53 mM day^–1^ at pH 11 (∼21.4 mM and 26.0 mM removal in 49 days), which is slower than past work which used the same sediment inoculum and acetate or lactate (2.14 mM day^–1^; Rizoulis et al., 2012; Bassil et al., 2015b).

At both pH 10 and 11, nitrate was partially reduced to nitrite (Figures 1C,D), whilst citrate was presumably fully oxidized to CO_2_ during anaerobic respiration, as no volatile fatty acid degradation products (e.g., acetate and other organic acids) were detected at any of the time points analyzed. At both pH 10 and 11, nitrite accumulated as the main product of nitrate reduction; peaking at 27.4 mM by day 35 at pH 10, and, 29.0 mM by day 49 at pH 11. Interestingly, between day 35 and 180 in the pH 10 and 11 systems, measurable nitrite removal was observed and the nitrite concentration had decreased by 30% at pH 10 and 24% at pH 11, by day 180 implying further denitrification had occurred. Given this, the proposed citrate degradation reactions for reduction of nitrate and nitrite are shown in Equations 1 and 2. Electron balance calculations, using standard electron equivalents show that approximately 0.04–0.05 mM electron equivalent of the citrate was consumed during reaction 1 and approximately 0.04 mM electron equivalent in reaction 2 (Supplementary Section 1 and Supplementary Tables 1, 2).

(1) $$C_{6}H_{5}O_{7}^{3-}+8NO_{3}^{-}+3H_{2}O\to 6HCO_{3}^{-}+8NO_{2}^{-}+5H^{+}$$

(2) $$3C_{6}H_{5}O_{7}^{3-}+16NO_{2}^{-}+2H_{2}O\to 18HCO_{3}^{-}+8N_{2}+OH^{-}$$

At pH 10, small quantities of both citrate and nitrate remained in solution by day 49, although these data points were within error of zero (Figure 1). It is clear that the electron equivalents generated via citrate oxidation, were consumed during both nitrate and nitrite reduction (Equation 1, Supplementary Section 1, and Supplementary Table 2). Electron balance calculations implied the remaining electron equivalents generated from oxidizing citrate were consumed by nitrite reduction at both pH 10 and pH 11 (Supplementary Table 2). Even though measured nitrite concentrations decreased, presumably by further reduction to N_2_, it seems likely that further denitrification was halted following removal of citrate and lack of alternative electron donor. Overall, the geochemical data show robust citrate degradation under nitrate reducing conditions at both initial pH 10 and 11.

Findings here are relevant to LLW disposal whilst also contributing to wider understanding of citrate degradation at high pH. Indeed, microbial citrate oxidation coupled to denitrification has been shown in a range of high pH environments, most notably in soda lakes at pH 10–10.5 (Switzer Blum et al., 1998; Boltyanskaya et al., 2007; Kim et al., 2007; Xu et al., 2007) and wastewater treatment at pH 10.25–11 (Sorokin et al., 2007; Fox et al., 2015). In the current work we confirm robust citrate metabolism during nitrate reduction at pH 11, the upper pH limit of previous observations. This implies nitrate reduction can play a significant role in the removal of citrate at pH conditions representative of those expected in low level radioactive waste disposal facilities.

### Fe(III)-Reducing Conditions

**FIGURE 2:**
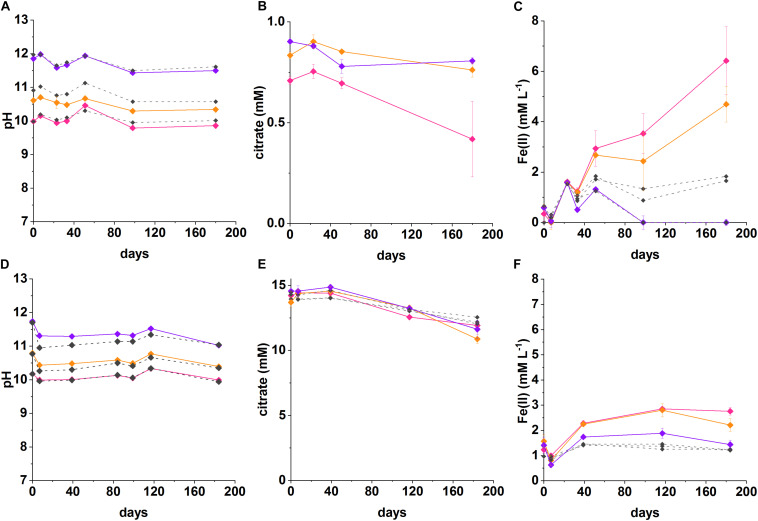
Recorded pH, Fe(II) and citrate measurements from anaerobic, high pH microcosms incubated for up to 200 days at 20°C in the dark (pH 10—pink, pH 11—orange, pH 12—purple, no-citrate **(A–C)** and sterile **(D–F)** control—dashed gray). **(A–C)** System with 1 mM citrate and 18 mM Fe(III), **(D–F)** 15 mM citrate and 15 mM Fe(III). The errors are shown and represent 1 SD on triplicate measurements.

Geochemical modeling of the Fe(III)-citrate system predicted differences in Fe(III)-citrate complexation as citrate concentrations changed (Supplementary Figure 2). Accordingly, to explore this further, two Fe(III)-reducing experiments were run with 1 mM (low) and 15 mM (high) citrate concentrations with data presented in Figure 2.

In the low citrate microcosms the pH was essentially constant during Fe(III) reduction at pH 10 (initial pH 10; final pH 9.9) and was slightly acidified for the initial pH 10.6 system (final pH of 10.3). There was no evidence for any pH change at pH 12. In terms of Fe(III)-reduction, in the pH 10 and pH 11 incubations, a darkening in color of the solids was observed in the inoculated experiments compared to the ferruginous, non-microbially active controls. This suggests reduction of Fe(III) to Fe(II), as Fe(II)-bearing minerals are typically darker in color (Supplementary Figure 3; Burke et al., 2005; Byrne et al., 2015). To further quantify the extent of Fe(III)-reduction, 0.5 N HCl extractions were performed which showed clear ingrowth of Fe(II) into the microcosm slurry. Here, maximum Fe(II) concentrations in slurries of 6.4 ± 1.3 mM at pH 10, and 4.7 ± 0.7 mM at pH 10.6 were measured after 180 days. The concentrations of Fe(II) measured in inoculated microcosms were significantly higher than in the no-citrate controls (Figure 2C). Although these 0.5 N HCl extractions provided a clear indication that Fe(II) was being produced in the biologically active microcosms, it is noted that they may have underestimated the total Fe(II) concentration within the microcosm as some Fe(II) may have been incorporated into magnetite which is poorly soluble under the 0.5 N HCl extractions (Sidhu et al., 1981). In these low citrate systems, the maximum citrate removal measured over 180 days was 0.30 ± 0.2 mM at pH 10 and 0.14 ± 0.04 mM at pH 11. These values were converted into electron equivalents for the system and used in the electron balance assessment (Table 1 and Supplementary Table 1).

**TABLE 1:** Electron balance assessment for Fe(III)-reducing microcosms.

	Citrate oxidized eeq mM	Fe(III) reduced eeq mM
**Low citrate [1 mM citrate: 18 mM Fe(III]**		
pH 10	5.2 ± 3.0	6.4 ± 1.3
pH 11	2.5 ± 0.8	4.7 ± 0.7
pH 12	1.6 ± 0.9	0
**High citrate [15 mM citrate: 15 mM Fe(III)]**		
pH 10	41 ± 6	2.9 ± 0.2
pH 11	39 ± 9	2.8 ± 0.3
pH 12	53 ± 12	1.9 ± 0.4

In the low citrate system with 1 mM citrate and 18 mM Fe(III) as ferrihydrite, geochemical modeling predicted the “free” citrate concentrations in solution as 0.004 mM at pH 10, 0.06 mM at pH 10.6, and 0.99 mM at pH 12. This was due to the reduced complexation of Fe(III) as pH increased, with the dominant Fe(III)-citrate complex {[Fe_2_(Cit)_2_(OH)_2_]^2–^]} predicted to form at a concentration of 0.5 mM at pH 10, 0.4 mM at pH 10.6, 0.007 mM at pH 12. The residual Fe(III) was speciated by the model as ferrihydrite. In these low citrate systems, citrate and Fe(III) were added in stoichiometric balance and assuming complete citrate oxidation to CO_2_ coupled to the reduction of Fe(III) to Fe(II), i.e, 1 mM citrate to 18 mM Fe(III):

(3) $$C_{6}H_{5}O_{7}^{3-}+18Fe^{3+}+11H_{2}O\to 18Fe^{2+}+6HCO_{3}^{-}+21H^{+}$$

Electron balance assessments for the low citrate experiment, showed that the citrate removed was coupled to Fe(III) reduction in a ratio of 0.8 at pH 10 and 0.5 at pH 10.6 (Table 1). Both values are within error of each other and approach the expected ratio of 1 for the stoichiometric reaction (Equation 3). These electron balance calculations revealed that slightly more Fe(III) was reduced than citrate oxidized, which suggested that there was an additional source of electron donor in the system. Candidates include the natural organic matter in the sediment (total organic carbon content of approximately 0.9%; Burke et al., 2012). Indeed, a small amount of Fe(II) ingrowth was observed in the no citrate controls (Figure 2C), confirming the presence of residual low level electron donor in the system. Nevertheless, Fe(II) ingrowth in the inoculated, low-citrate microcosms was still significantly greater than in the no-citrate controls, which confirmed that citrate oxidation fuelled the majority of observed Fe(III) reduction.

Under initial conditions, the percentage of the added Fe(III) predicted to speciate as the [Fe_2_(Cit)_2_(OH)_2_]^2–^ citrate complex at pH 10 was 3.3%, at pH 10.6 was 2.6% and at pH 12 was 0.05%. The measured percentages of added Fe(III) that was reduced were: 35% at pH 10 and 27% at pH 10.6, no Fe(II) ingrowth was detected in the pH 12 samples. The amount of Fe(III) that was reduced in the pH 10 and pH 10.6 experiments was 10 times greater than the amount of [Fe_2_(Cit)_2_(OH)_2_]^2–^ predicted to form, which suggests that both the soluble [Fe_2_(Cit)_2_(OH)_2_]^2–^ and solid ferrihydrite pools were reduced with the solid ferrihydrite presumably able to re-equilibrate, and solubilize, by complexation with free citrate. The importance of citrate complexation in facilitating Fe(III) reduction in these experiments is further supported by the fact that no Fe(II) was measured in the pH 12 experiment where only 0.05% of the added Fe(III) was predicted to be complexed by citrate. This is in agreement with observations that the reduction of insoluble Fe(III) is extremely challenging at pH ≥ 11 due to the lower energy yield available (Rizoulis et al., 2012).

In the high citrate system 15 mM citrate and 15 mM ferrihydrite were added to the microcosms. The geochemical model (Supplementary Figure 2) predicted significant Fe(III)-citrate complexation as the [Fe_2_(Cit)_2_(OH)_2_]^2–^ species with concentrations of 2.7 mM at pH 10; 1.9 mM at pH 11; 0.4 mM at pH 12. In addition, modeling predicted the remaining citrate would be present dominantly as the free citrate (Cit^3–^) species with concentrations of 9.5 mM at pH 10; 11.3 mM at pH 11; and 14.3 mM at pH 12.

In the inoculated systems, similar to the low citrate experiments, a darkening was observed after 1 week of incubation from ferruginous to dark-brown. This was not seen in the heat-sterilized controls which remained rust-colored. Indeed, after 40 days, ingrowth of Fe(II) was detected by 0.5 N HCl sediment extractions in all inoculated systems at initial pH values of 10.2, 10.8 and even at pH 11.7. The maximum Fe(II) concentrations detected over the duration of the experiment were: in the pH 10 system 2.9 ± 0.07 mM; in the pH 10.6 system 2.8 ± 0.3 mM; and in the pH 11.7 system 1.9 ± 0.4 mM. The final pH values were relatively constant at pH 10.0, pH 10.6 and pH 11.5, respectively. In addition to Fe(II) ingrowth, the measured citrate removal after 180 days was: in the pH 10.2 system 2.3 ± 0.4 mM; in the pH 10.8 system 2.2 ± 0.5 mM; in the pH 11.7 system 2.9 ± 0.7 mM.

The electron balance assessment for the citrate oxidation/Fe(III) reduction for these high citrate systems is provided in Table 1, and show the systems were electron acceptor limited. Here, for the initial experimental concentrations of 15 mM citrate and 15 mM Fe(III), oxidation of all added citrate would generate 18 times more electron equivalents than could be consumed by reduction of all added Fe(III). The experimental data for the pH 10.2, 10.8, and 11.7 experiments showed citrate oxidation generated an average of 44.3 ± 16 mM electron equivalent whilst Fe(III) reduction consumed an average of 2.3 ± 0.6 mM electron equivalent (experiments at each pH were all within error of each other; Table 1). This gave a ratio of electron equivalents generated to electron equivalents consumed of approximately 19 ± 8. Indeed, 39–53 mM electron equivalents, were generated during the oxidation of citrate whilst consumption of only 1.9–2.9 mM electron equivalents was calculated from measured Fe (II) ingrowth (Table 1). As previously mentioned for the low citrate systems, the concentration of Fe(II) produced during the reduction reaction may have been underestimated as some of the Fe(II) was presumably incorporated into magnetite which is poorly extractable in 0.5 N HCl (Sidhu et al., 1981). This is consistent with the final sample point where the mineral phases were magnetic and black suggesting significant magnetite ingrowth (Supplementary Figure 3). However, even if all added 15 mM Fe(III) were reduced, there would still have been an excess of 24–38 electron equivalents generated according to the calculations based on citrate removal.

Another possible sink for the excess electron equivalents produced during citrate oxidation could have been other biogeochemical reactions within the sediment, e.g., fermentation. Data from the pH 10 microcosms showed acetate ingrowth of 0.7 ± 0.2 mM (high citrate) and 0.3 ± 0.2 mM (low citrate) in the day 180 samples and no acetate in the parallel controls. This suggested some citrate fermentation was occurring in these microcosms. However, only trace quantities of acetate (<0.05 mM) were detected in the other microcosms. It is also possible that citrate underwent incomplete degradation to form other metabolites that were not detectable by IC analysis. In addition, other processes such as sorption could be contributing overall citrate removal from solution, and therefore toward a slight overestimation of citrate oxidation in the electron balance calculations. Examining the autoclaved controls revealed only 12–16% of added citrate was estimated to have sorbed to microcosm solids. It remains unclear how excess electron equivalents from citrate oxidation, in the high citrate systems, are being utilized in these highly complex Fe(III)-reducing systems.

In the inoculated experiments at pH 10.2, 10.8, and 11.7 the percentages of Fe(III) reduced were 18, 19, and 13%, respectively. Geochemical modeling predicted percentages of added Fe(III) that would be speciated as the [Fe_2_(Cit)_2_(OH)_2_]^2–^ complex at pH 10, 11 and 12 were 18, 13, and 2.3%, respectively. Since experimental data show that more Fe(III) is reduced than is predicted to be soluble under initial conditions, it suggests that some of the solid Fe(III) was bioavailable. The increase in bioavailability of the solid Fe(III) was presumed to result from re-equilibration of free citrate with ferrihydrite to form more of the soluble [Fe_2_(Cit)_2_(OH)_2_]^2–^ complex, and/or, the direct reduction of solid Fe(III).

The bioreduction solids which had ingrown to the microcosms in low- and high-citrate systems were tested at day 50 using a magnet. Here, only the inoculated microcosms where dark mineral precipitates were visible responded to the magnet, these were the pH 10 and 10.6 microcosms in the low citrate system, and, pH 10.2, 10.8, and 11.7 microcosms in the high citrate system (Supplementary Figure 3). XRD analysis of selected samples from both the low- and high-citrate systems was attempted and in all samples the detection limit was too low due to the high background from e.g., calcite in the sediment inoculum.

**FIGURE 3:**
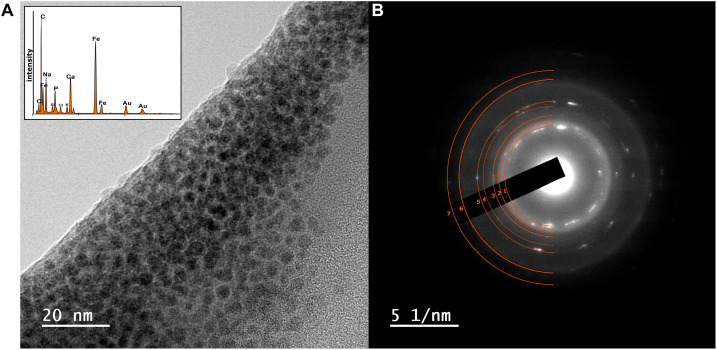
**(A)** TEM image of nanoparticulate magnetite with EDAX data inset showing Fe peaks of high relative intensity **(B)** corresponding SAED pattern from a sample taken from the high-citrate Fe(III)-reducing microcosm experiments at initial pH 11.7. The SAED pattern **(B)** from the magnetite particles in **(A)**, have the index for magnetite overlaying the pattern (d-spacing values are provided in Supplementary Figure 5 and Supplementary Table 3; Sun et al., 2017).

At this stage, to further characterize the structure, particle size, and morphology of the Fe in the sample, TEM analysis using EDAX and SAED was performed on selected samples from the high citrate system, from pH 10.2, 10.8, and 11.7 experiments (data for the pH 11.7 sample are shown in Figure 3, data for pH 10.2 and 10.8 samples are shown in Supplementary Figure 5). Here, transmission electron microscopy allowed identification of Fe rich nanoparticles with particle sizes of approximately 2–5 nm in diameter and with similar morphology to nanoparticulate magnetite (Roberts et al., 2017). The selected area electron diffraction pattern (Figure 3) confirmed the presence of nanoparticulate magnetite with the index for magnetite overlaying the pattern from the experimental sample (Sun et al., 2017).

Overall, these data provide compelling evidence for development of Fe(III)-reduction in the microcosm with initial pH 11.7; with darkening of sample, increase in 0.5 N HCl extractable Fe(II), response of the reduced sample to a magnet and transmission electron microscopy coupled to selected area electron diffraction, all consistent with nanoparticulate magnetite formation. These observations for Fe(III)-reduction exceeds the highest pH values of 11 reported for Fe(III) reduction for pure culture (Ye et al., 2004; Pollock et al., 2007) and 10.5–10.8 for mixed microbial cultures of the type used in the current work (Stewart et al., 2010; Rizoulis et al., 2012; Fuller et al., 2014). Interestingly, this suggests that citrate may play a key role in enabling high pH Fe(III)-bioreduction by enhancing Fe(III)-availability to cells through chelation. Indeed, in these experiments, where little bioavailable (soluble) Fe(III) was present in the pH ∼12 systems, little or no Fe(III) bioreduction was observed. This was despite the presence of a substantial pool of bioavailable citrate in each inoculated microcosm, as [Cit]^3–^ [Fe_2_(Cit)_2_(OH)_2_]^2–^ or [Ca(Cit)]^–^ (Francis and Dodge, 1993; Szecsody et al., 2007; Lensbouer and Doyle, 2010; Jeen and Hyun, 2015). Bioavailability of the Fe(III) is therefore the most likely factor which controlled bioreduction in these systems.

The solubilization of solid Fe(III) by chelation is a strategy thought to be employed by some dissimilatory Fe(III)-reducers to solubilize Fe(III) (Weber et al., 2006). Indeed, solubilizing Fe(III) eliminates the requirement for direct contact with the mineral during Fe(III) reduction. Chelating agents are known to stimulate Fe(III) reduction, for example, some dissimilatory Fe(III)-reducers, such as *Geothrix fermentans* and *Shewanella alga* BrY, secrete them during Fe(III) reduction (Nevin and Lovley, 2002a, b). Furthermore, use of soluble ferric-citrate is known to enhance utilization of Fe(III) compared to ferrihydrite (Schröder et al., 2003; Bird et al., 2011), including at high pH (Rizoulis et al., 2012).

These experiments have illustrated the complexity of high pH, Fe(III)-reducing systems and challenges faced with their measurement/characterization. Some of the various potential biogeochemical factors that may influence the fate of citrate in a repository were highlighted, and here these included: the formation of bio-minerals, adsorption reactions and impacts from other biological activity taking place simultaneously in sediment systems. Overall, data here provides strong evidence for robust Fe(III) reduction at pH values up to pH 11.7 with citrate acting as both an electron donor and Fe(III) complexant thus potentially enhancing Fe(III) bioavailability. Results here confirm that in a repository setting, the consumption of citrate coupled to Fe(III) reduction will likely lead to reduction in citrate concentration in wastes and can contribute toward establishing a reducing environment favorable to radionuclide retention in solids.

### Sulfate Reducing Conditions

**FIGURE 4:**
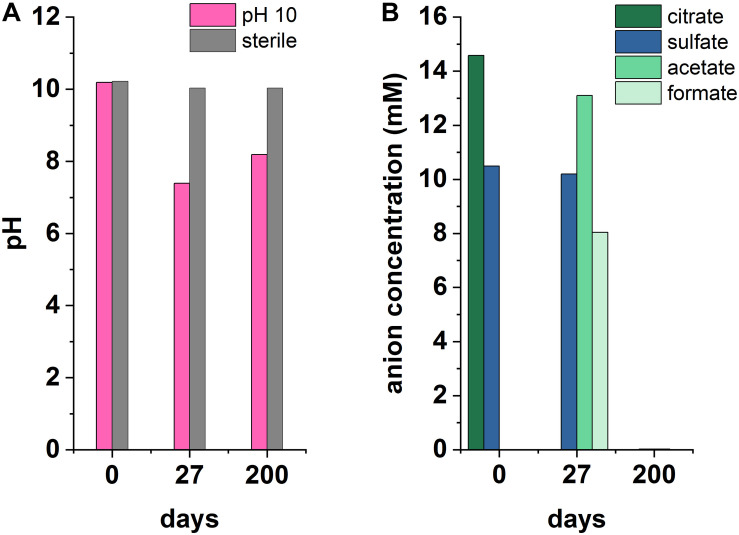
Recorded pH **(A)** and anionic species **(B)** measurements from an anaerobic, pH 10 microcosm under sulfate-reducing conditions, incubated for 200 days at 20°C in the dark.

Microcosms were set up to test sulfate reduction in sediment inoculated experiments at pH 10, 11, and 12. Overall, sulfate reduction only occurred in one outlying microcosm of a triplicate set at pH 10 (Figure 4). Here, a decrease in pH from 10.2 to 7.4 and complete removal of citrate occurred by day 27. Simultaneously, acetate and formate had accumulated to 13.1 and 8 mM, respectively. Acetate and formate were removed from solution by the 200 day end point. Interestingly, sulfate reduction occurred between day 27 and 200 with complete removal of sulfate by 200 days which was accompanied by a blackening of sediments and a characteristic hydrogen sulfide smell at the end-point. Overall this suggests a sporadic development of sulfate reduction, and only in the pH 10 experiment. Additionally, the microcosm which became sulfidic clearly suggests citrate fermentation occurred as the initial degradation step in this sulfate reducing microcosm. Here, the pH was acidified to pH 7.4 which is more favorable to microbial sulfate reduction (Rizoulis et al., 2012). Latterly, acetate and formate were detected and sulfate, acetate and formate were completely removed by day 200. This suggests that these fermentation products were used as electron donors to fuel sulfate reduction. It is interesting that fermentation only appears to have occurred in this single microcosm, and not in the fermentation experiment (Supplementary Figure 1), and, reasons for this remain unclear. One possibility is heterogeneity within the inoculum introduced these organisms by chance.

Although sulfate reduction was only observed in one microcosm, these results highlight the impact that environmental heterogeneity has in heterogeneous systems. Results here imply that the onset of sulfate reduction, coupled to citrate biodegradation, is possible and may be favored by localized environments where less-alkaline pH prevails. This also leads to the potential for local zones of citrate fermentation promoting acidification and sulfate reduction in wasteforms. Importantly, microbial sulfate reduction generates both reducing conditions and ingrowth of sulfide which can react with metal cations causing reduction of soluble, oxic forms of metal ions, and/or precipitation of insoluble metal-sulfide minerals. Indeed, precipitation of sulfide minerals may remove contaminants including Ni, Cd, Zn, Cu, Cr (Gadd, 2000, 2004; Krumholz et al., 2003; Kuippers et al., 2018) and radionuclides including U and Tc (Lovley and Phillips, 1992; Lloyd et al., 1998; Beyenal et al., 2004).

### Microcosm Microbial Communities

**FIGURE 5:**
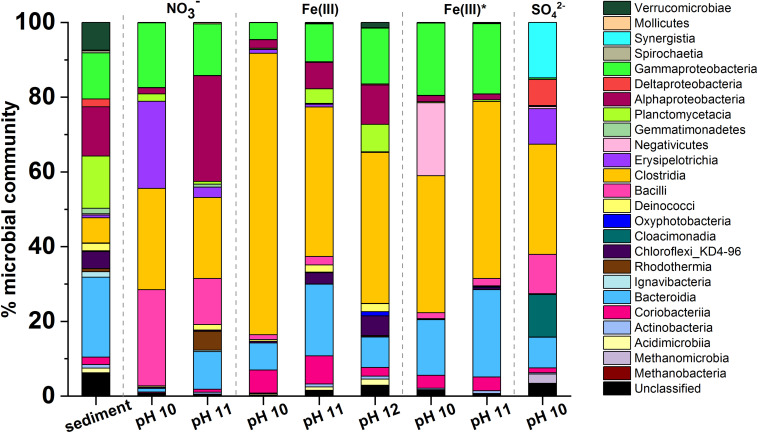
Microcosm microbial community profiles by phylogenetic class. In all incubations there was an increase in the relative abundance of Gram-positive Firmicutes. Clostridia was the dominant class in most samples. Other key classes observed included Gammaproteobacteria, Alphaproteobacteria, Bacteroidia, and Bacilli. The sulfate-reducing microcosm had a significantly different population when compared to the other microcosms and this included Synergistia, Deltaproteobacteria, and Cloacimonadia.

PCR-based high-throughput 16S rRNA gene sequencing was used to analyze communities in biologically active sample (the sequencing data have been uploaded to the NCBI Sequence Read Archive; http://www.ncbi.nlm.nih.gov/sra/ under the project accession number: PRJNA691128). Data in Figure 5, show the initial sediment inoculum contained 562 operational taxonomic units (OTUs) and diversity was reduced by the end of each experiment Alpha-diversity plots (Supplementary Figure 7) showed that the diversity across samples, from highest to lowest, was in the order: Fe(III)-reducing pH 12 > Fe(III)-reducing pH 11 > nitrate-reducing pH 11 > Fe(III)-reducing pH 10 > nitrate-reducing pH 10 > sulfate -reducing pH 10. Here, diversity decreased at lower pH levels where biological activity was most intense, and well adapted individuals were able to grow more rapidly to colonize the niche.

The dominant phyla in the starting inoculum were Gram-negative Proteobacteria (27%), Bacteroidetes (20%) and Planctomycetes (25%; Figure 5). A profound change in community composition was observed across all microcosms, and end point samples were all distinct from the starting inoculum. In all samples an increase in the relative abundance of Gram-positive Firmicutes from 2% in the inoculum to > 30% was observed. In addition, the experimental controls included throughout indicated that contamination of samples was negligible (Supplementary Figure 6).

Under nitrate-reducing conditions at pH 10, end point samples were dominated by Firmicutes (78%) followed by Proteobacteria (18%). At pH 11 Proteobacteria (43%) and Firmicutes (40%) also dominated. At the genus level, in the pH 10 and 11 samples, members of the genus *Anaerobacillus* dominated (48 and 12%; Supplementary Figure 7). Notably, a close relative of *Anaerobacillus alkalilacustris* (99% identity match), a known alkaliphile capable of oxidizing organics and reducing nitrate, was present (Zavarzina et al., 2009; Bassil and Lloyd, 2018). The pH 11 sample also contained a novel organism most closely related to *Symbiobacterium* (92% match; 13% of sequences); members are known to reduce nitrate at alkaline pH.

In all Fe(III)-reducing experiments Firmicutes dominated at all pH values tested (47–77%). A close relative of *Dethiobacter alkaliphilus* (98% match) was one of the most dominant organisms in all samples from both the high- and low- citrate systems (20–33%; Supplementary Figure 8). Interestingly, this is a haloalkaliphilic and sulfidogenic microbe which is known to have multiple *c-*type cytochromes which facilitate extracellular electron transport to reduce insoluble Fe(III) (Wrighton et al., 2011). In addition, a relative of the *Tindallia* genus (93% sequence match) was dominant in pH 10 samples of high citrate experiments (37%), and was also present in the low citrate experiments (5%). Known alkaliphillic members of this genus are capable of citrate fermentation and Fe(III) reduction (Alazard et al., 2007); this supports the geochemical data which suggested that some fermentation may be occurring in the Fe(III)-reducing systems.

Firmicutes were also dominant in the sulfate-reducing sample (45%), followed by Synergistetes (19%), and Cloacimonetes (15%). By genus (Supplementary Figure 9), the three most dominant organisms were fermentative bacteria: a close relative of *Cloacibacillus* (91% match, 15% of sequences), an uncultured microbe affiliated with the family *Cloacimonadacea* (11% of sequences), and *Trichococcus alkaliphilus* (100% match; 10% of sequences). These organisms all ferment organic acids to produce CO_2_, H_2_ and/or short chain fatty acids, including acetate and formate (Looft et al., 2013; Dai et al., 2018; Dyksma and Gallert, 2019). The sample also contained known sulfate reducers (>9% of sequences detected, collectively) such as *Desulfomicrobium baculatum* and *Desulfotomaculum acetoxidans.* Here, the microbial community present in this sulfate reducing sample supports the suggestion that fermentative organisms degraded citrate to produce acetate and formate (Figure 4), which were then oxidized during sulfate reduction. This mechanism has been previously observed by Gámez et al. (2009) and Stams et al. (2009); the latter also identified a member of the *Trichococcus* genus as a key citrate-fermenter. Interestingly, methanogenic Euryarchaeota (4%) were also detected in this sample, the largest portion of which were identified as *Methanosarcina spelaei* (100% match; 3% of sequences detected), this organism can grow using H_2_/CO_2_ and organic by-products formed during citrate degradation (Ganzert et al., 2014). This suggested that methanogenesis may have been taking place in this microcosm, although this was not confirmed as methane measurements were not performed during these experiments.

## Conclusion

Citrate was fully degraded at pH 10 and pH 11, with nitrate as TEA. In Fe(III)-reducing systems, citrate was partially degraded at pH 10–11.7. Fe(III)-reduction in the pH 11.7 system is to our knowledge the highest pH Fe(III)-reducing system reported and we postulate this was a function of complexation of Fe(III) by citrate, even at pH 11.7 in the high citrate experiment, enabling bioreduction of the soluble Fe(III)-citrate species. Finally, sulfate reduction was sporadic and only developed at pH 10 and then only after significant acidification from fermentation. Overall, these findings provide evidence that in a LLW repository setting, citrate will be degraded and removed from solution and will promote development of reducing conditions, thus preventing the mobilization of metal contaminants.

Research regarding anaerobic microbial metabolism at high pH has been gaining momentum in recent years, especially in context of cementitious repositories where the impacts of microbial colonization are considered significant (Williamson et al., 2013, 2014, 2015; Bassil et al., 2015a, b; Charles et al., 2015; Rout et al., 2015a, b; Durban et al., 2018; Nixon et al., 2018; Mijnendonckx et al., 2020). Previous work has confirmed anaerobes will colonize wastes; metabolizing the waste components by fermentation or a cascade of terminal electron accepting processes (Rizoulis et al., 2012). Overall, this work is significant as the rate and extent of microbial citrate degradation, at high pH, is shown for the first time. This information can now be used to underpin assumptions made in Environmental Safety Case near field models, and the development of waste acceptance criteria based upon them, for high pH cementitious repositories.

## Data Availability Statement

The datasets presented in this study can be found in online repositories. The names of the repository/repositories and accession number(s) can be found below: NCBI SRA, PRJNA691128.

## Author Contributions

NB (primary investigator): experimental design, sample collection, microcosm experiment set-up, pH and Eh measurements, ferrozine assay, DNA extraction, geochemical modeling, data processing and interpretation, and manuscript writing. JL: experimental design, data interpretation, and manuscript review. JS: support and contextualization for experimental design, support with geochemical modeling, and manuscript review. FT: support and contextualization for experimental design, and manuscript review. HB: TEM support. CB: DNA sequencing. KM: experimental design, data interpretation, and manuscript drafting and review. All authors contributed to the article and approved the submitted version.

## Conflict of Interest

The authors declare that this study received funding from Low Level Waste Repository Ltd. The funder had the following involvement with the study: support and contextualization for experimental design, manuscript review. The funder was not involved in the collection, analysis and interpretation of data, or, the decision to submit it for publication. FT was employed by company Low Level Waste Repository Ltd. The remaining authors declare that the research was conducted in the absence of any commercial or financial relationships that could be construed as a potential conflict of interest.
