# Supplementary material for: Microbial Degradation of Citric Acid in Low Level Radioactive Waste Disposal: Impact on Biomineralization Reactions
Source: Front Microbiol. 2021 Apr 28;12:565855. doi: 10.3389/fmicb.2021.565855 (PMC8114274; doi:10.3389/fmicb.2021.565855)
Supplement: Supplementary file 1 [file Data_Sheet_1.docx]

Supplementary Material

# Supplementary information

**Nitrate reducing experiment electron balance information**

Experimental values for citrate oxidation and nitrate reduction were converted to standard electron equivalents (eeq mol^-1^; Table S1), and these were used to perform an electron balance assessment (Table S2). Calculations predicted that oxidation of 5 mM citrate would generate 0.09 electron equivalents, whilst reduction of 30 mM nitrate would consume 0.06 electron equivalents – a predicted 3:2 oxidation/reduction ratio. The electron balance assessment for experimental data (Table S2) indicates that at pH 10 the oxidation of citrate coupled to reduction of nitrate to nitrite was in an oxidation/reduction ratio of 2:1 ratio. Here, half of the electron equivalents generated from citrate oxidation were consumed during nitrate reduction. In the pH 10 experiments, the electron balance assessment suggests further denitrification had taken place as the remaining half of electron equivalents generated from citrate oxidation were accounted for by the nitrite reduction reaction. At pH 11 the electron balance assessment suggests that the oxidation of citrate coupled to the reduction of nitrate was in an oxidation/reduction ratio of 9:5. The remaining electron equivalents generated during citrate oxidation were also accounted for during nitrite reduction, which occurred in an oxidation/reduction ratio of 9:4.

# Supplementary Figures and Tables

The figures and tables are listed in the order in which they are mentioned in the research article.





**Figure S1** Results over 200 days from citrate fermentation experiment (15 mM citrate added, no electron acceptor), showing pH measurements (A) and measured citrate concentrations (B). Legend: pink - pH 10, orange - pH 11, purple- pH 12, dashed grey - sterile controls.

Table S1 Standard eeq mol^-1^ values used in electron balance assessment calculations calculated from standard half equations (McCarty, 2006)

| Reaction: | **eeq mol^-1^** |
| --- | --- |
| Citrate 🡪 CO_2_ | 18 |
| NO_3_^-^ 🡪 NO_2_^-^ | 2 |
| NO_2_^-^ 🡪 $\frac{1}{2}$N_2_ | 1.5 |
| Fe(III) 🡪 Fe(II) | 1 |

**Table S2** NO_3_^-^-reducing experiment electron balance assessment

|  | **Citrate oxidized**  eeq mM | **Nitrate reduced**  eeq mM | **Nitrite reduced**  eeq mM |
| --- | --- | --- | --- |
| pH 10 | 0.08 ± 0.001 | 0.04 ± 0.003 | 0.04 ± 0.01 |
| pH 11 | 0.09 ± 0.0004 | 0.05 ± 0.0006 | 0.04 ± 0.001 |
| pH 12 | 0.01 ± 0.0004 | 0 | 0 |



**Figure S2** PHREEQC modelling outputs for two different Fe(III)-reducing microcosm systems; (A)1 mM citrate:18 mM Fe(III), (B) 15 mM citrate: 15 mM Fe(III).


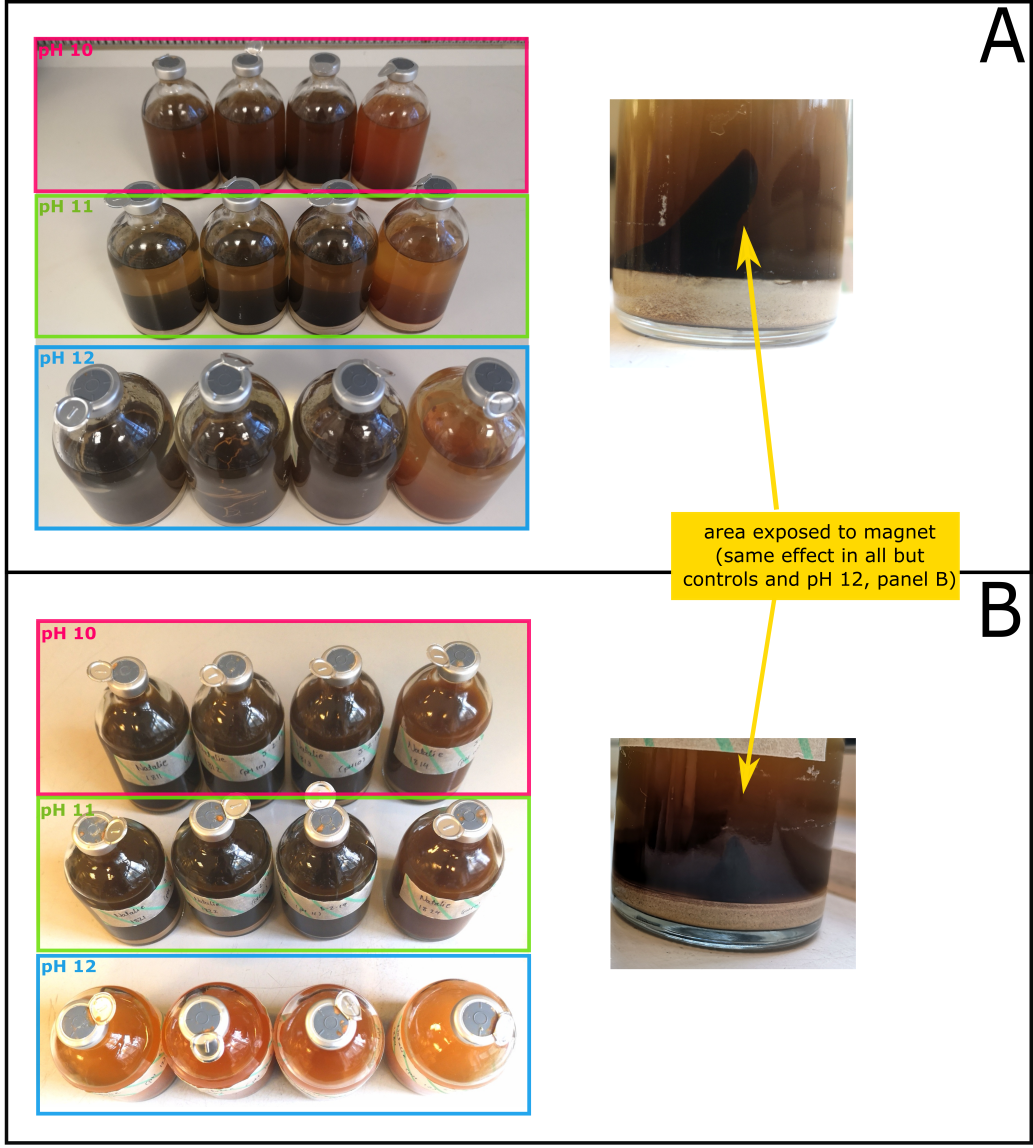


**Figure S3** Images showing Fe(III)-reducing microcosm colour change and response to magnet. In each set, the right most bottle is the control. Panel A: 15 mM citrate and 15 mM Fe(III) concentrations, Panel B: 1 mM citrate, 18 mM Fe(III).


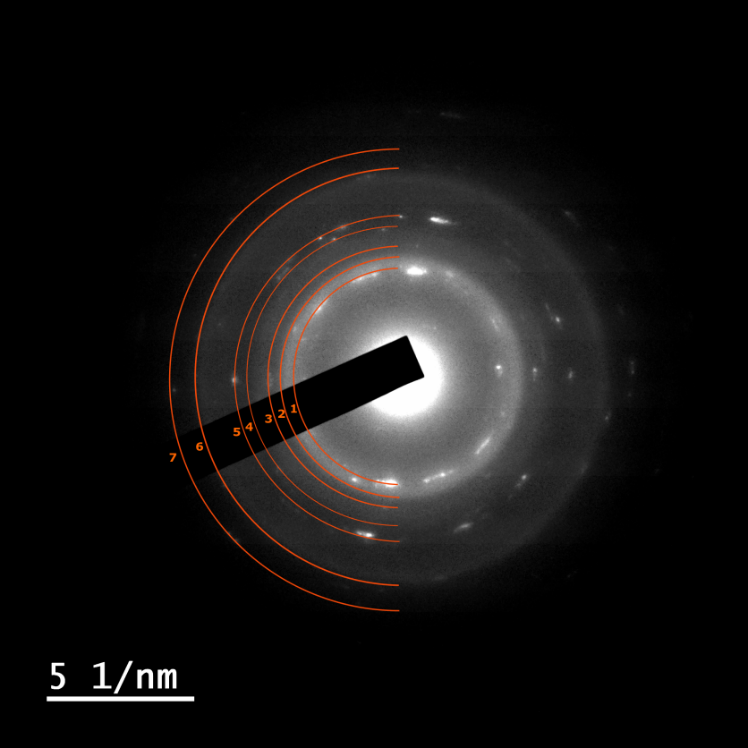


**Figure S4** SAED pattern obtained from pH 11.7 sample labelled (as in manuscript), corresponding d-spacing values are in Supplementary Table 1 below

**Table S3** D-space values for sample compared to magnetite standard d-spacings (Sun et al., 2017)

| **Label on diagram** | **Sample** (Å) | **Magnetite standard d-space** (Å)* |
| --- | --- | --- |
| 1 | 2.90 | 2.97 |
| 2 | 2.54 | 2.53 |
| 3 | 2.00 | 2.1 |
| 4 | 1.74 | 1.71 |
| 5 | 1.48 | 1.48 |
| 6 | 1.28 | 1.28 |
| 7 | 1.25 | 1.21 |


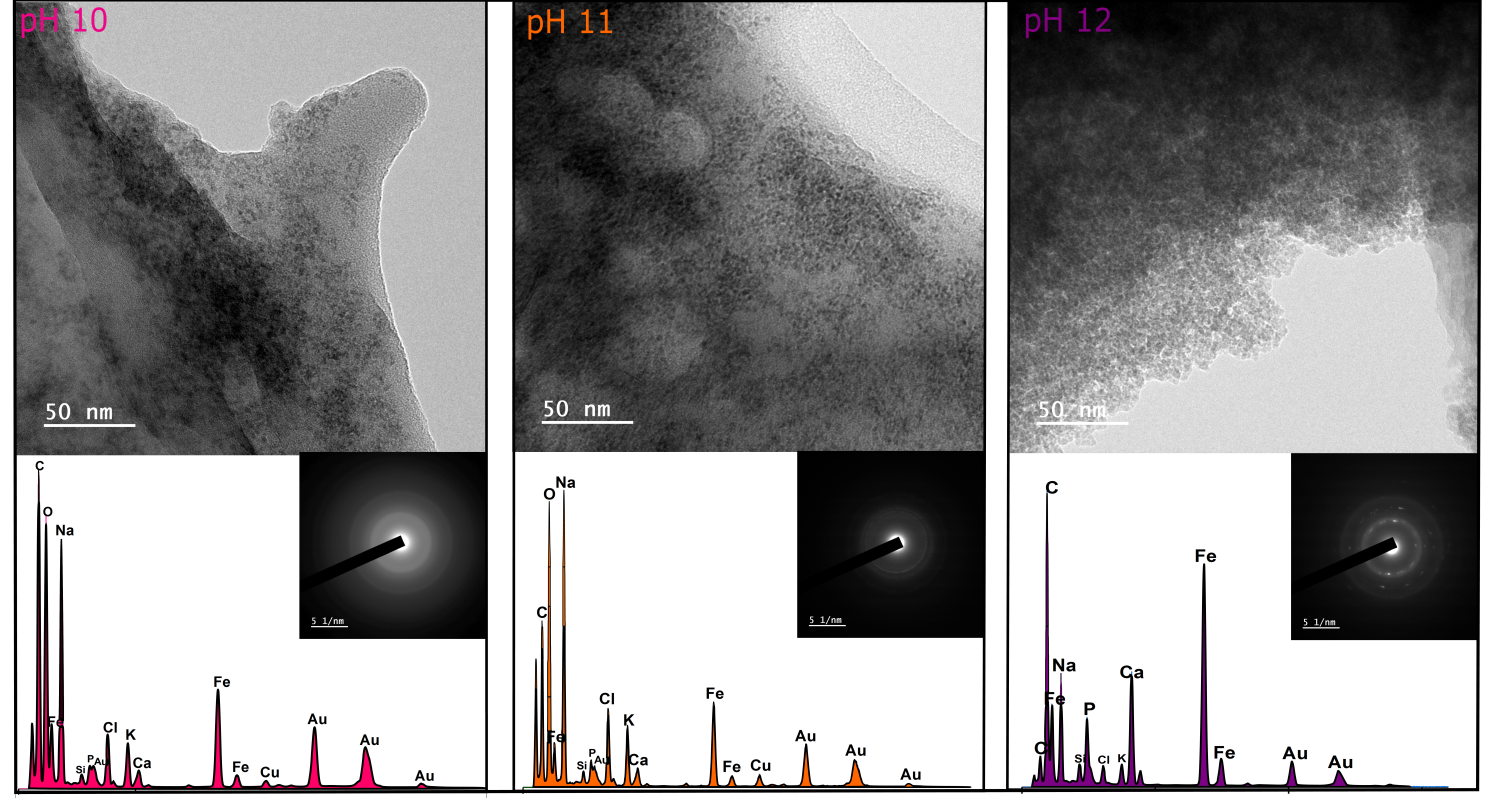


**Figure S5** TEM images of samples from the high-citrate Fe(III)-reducing experiments at pH 10 (10.2) pH 11 (10.8) and pH 12 (11.7). EDAX data are included below the image for each sample, with the SAED patterns for Fe(III) rich areas inset. Legend: pink – pH 10, orange – pH 11, purple – pH 12.

**Table S4** Collation of information regarding high pH Fe(III)-reduction recorded in the literature

| **Reference** | **pH** | **Source** | **Microorganism(s)** |
| --- | --- | --- | --- |
| Pollock et al., 2007 | 11 | Salt-Flat Sediment | *Bacillus sp (SFB)* |
| Ye et al., 2004 | 11 | Borax leachate ponds | *Alkaliphilus metalliredigens* |
| Rizoulis et al., 2012 | 10.8 | Harpur Hill | mixed culture |
| Zakharyuk *et al.*, 2017 | 10.7 | Soda lake (Russia) | *Alkaliphilus namsaraevii* |
| Switzer Blum et al., 1998 | 10.5 | Mono Lake, California | *Bacillus arsenicoselenatis* etc. |
| Stewart *et al.*, 2010 | 10.5 | Chromite Ore Processing Residue | soil consortium |
| Gorlenko *et al.*, 2004 | 10.4 | Mono Lake, California | *Anaerobranca californiensis* |
| Zhilina *et al.*, 2009 | 10.2 | Soda lake | *Natronincola ferrireducens, Natronincola peptidovorans* |





**Figure S6** The α-diversity plot showing number of operational taxonomic units (OTUs) in the background sediment and sample end points. Legend notes: * high-citrate systems, **low-citrate systems





**Figure S7** Data showing the microbial community structure at Genus level of end point samples from the nitrate reducing experiments at pH 10 and pH 11, compared to the starting inoculum





**Figure S8** Data showing the microbial community structure at Genus level of end point samples from the Fe(III)-reducing experiments from both high- and low- citrate systems (left to right: high-citrate pH 10, 11, 12; low-citrate pH 10, 11), compared to the starting inoculum.





**Figure S9** Data showing the microbial community structure at Genus level of the end point sample from the Sulfate-reducing pH 10 microcosm compared to the starting inoculum.

**References**

Gorlenko, V., Tsapin, A., Namsaraev, Z., Teal, T., Tourova, T., Engler, D., et al. (2004). Anaerobranca californiensis sp. nov., an anaerobic alkalithermophilic, fermentative bacterium isolated from a hot spring on Mono Lake. *Int. J. Syst. Evol. Microbiol.* 54, 739–743. doi:10.1099/ijs.0.02909-0.

McCarty, P. L. (2006). Thermodynamic Electron Equivalents Model for Bacterial Yield Prediction: Modifications and Comparative Evaluations. *Biotechnol. Bioeng.* 97, 377–388. doi:10.1002/bit.

Pollock, J., Weber, K. A., Lack, J., Achenbach, L. A., Mormile, M. R., and Coates, J. D. (2007). Alkaline iron(III) reduction by a novel alkaliphilic, halotolerant, Bacillus sp. isolated from salt flat sediments of Soap Lake. *Appl. Microbiol. Biotechnol.* 77, 927–934. doi:10.1007/s00253-007-1220-5.

Rizoulis, A., Steele, H. M., Morris, K., and Lloyd, J. R. (2012). The potential impact of anaerobic microbial metabolism during the geological disposal of intermediate-level waste. *Mineral. Mag.* 76, 3261–3270. doi:10.1180/minmag.2012.076.8.39.

Stewart, D. I., Burke, I. T., Hughes-Berry, D. V., and Whittleston, R. A. (2010). Microbially mediated chromate reduction in soil contaminated by highly alkaline leachate from chromium containing waste. *Ecol. Eng.* 36, 211–221. doi:10.1016/j.ecoleng.2008.12.028.

Sun, S., Gebauer, D., and Cölfen, H. (2017). Alignment of Amorphous Iron Oxide Clusters: A Non-Classical Mechanism for Magnetite Formation. *Angew. Chemie - Int. Ed.* 56, 4042–4046. doi:10.1002/anie.201610275.

Switzer Blum, J., Burns Bindi, A., Buzzelli, J., Stolz, J. F., and Oremland, R. S. (1998). Bacillus arsenicoselenatis, sp. nov., and Bacillus selenitireducens, sp. nov.: Two haloalkaliphiles from Mono Lake, California that respire oxyanions of selenium and arsenic. *Arch. Microbiol.* 171, 19–30. doi:10.1007/s002030050673.

Ye, Q., Roh, Y., Carroll, S. L., Blair, B., Zhou, J., Zhang, C. L., et al. (2004). Alkaline anaerobic respiration: Isolation and characterization of a novel alkaliphilic and metal-reducing bacterium. *Appl. Environ. Microbiol.* 70, 5595–5602. doi:10.1128/AEM.70.9.5595-5602.2004.

Zakharyuk, A., Kozyreva, L., Ariskina, E., Troshina, O., Kopitsyn, D., and Shcherbakova, V. (2017). Alkaliphilus namsaraevii sp. nov., an alkaliphilic iron- and sulfur-reducing bacterium isolated from a steppe soda lake. *Int. J. Syst. Evol. Microbiol.* 67, 1990–1995. doi:10.1099/ijsem.0.001904.

Zhilina, T. N., Zavarzina, D. G., Osipov, G. A., Kostrikina, N. A., and Tourova, T. P. (2009). Natronincola ferrireducens sp. nov., and Natronincola peptidovorans sp. nov., new anaerobic alkaliphilic peptolytic iron-reducing bacteria isolated from soda lakes. *Microbiology* 78, 455–467. doi:10.1134/s0026261709040092.
